# Supplementary material for: Modelling and measuring complexity of traditional and ancient technologies using Petri nets
Source: PLoS One. 2022 Nov 29;17(11):e0278310. doi: 10.1371/journal.pone.0278310 (PMC9707756; doi:10.1371/journal.pone.0278310)
Supplement: S1 Appendix — Tables. Section A) initial markings and final markings of the subprocesses of the A. coranica model (Tables 1–8) and the O. schinzii model (Tables 9–16). Section B) Variables for the A. coranica and O. Schinzii models used to calculate the reachability graphs (Table 17). (DOCX) [file pone.0278310.s005.docx]

**S4 Appendix. Tables**

**Section a. Initial markings and final markings of the subprocesses of the A. coranica model (tables 1-8) and the O. schinzii model (Tables 9-16).**

Table 1 Minimum enabling marking and final marking for the places enabling or connecting (highlighted in grey) the Subprocess 1 Dig Bulbs with other subprocess of the A. coranica model with one available maker

| **Place** | **Initial marking** | **Final marking** |
| --- | --- | --- |
| Start1 | 1 | 0 |
| People available | 1 | 1 |
| Bulbs | 0 | 4 |
| Start5 | 0 | 1 |
| Digging sticks | 1 | 1 |
| Bulbs needed | 4 | 0 |

Table 2 Minimum enabling marking and final marking for the places enabling or connecting (highlighted in grey) the Subprocess 2 Collect Firewood and Branches with other subprocesses of the A. coranica model with one available maker

| **Place** | **Initial marking** | **Final marking** |
| --- | --- | --- |
| Start2 | 1 | 0 |
| People available | 1 | 1 |
| Collected Firewood | 0 | 4 |
| Collected Branches | 0 | 4 |
| Start3 | 0 | 1 |
| Firewood needed | 4 | 0 |
| Branches needed | 4 | 0 |

Table 3 Minimum enabling marking and final marking for the places enabling or connecting (highlighted in grey) the Subprocess 3 Light Fire with other subprocesses of the A. coranica model with one available maker

| **Place** | **Initial marking** | **Final marking** |
| --- | --- | --- |
| Start3 | 1 | 0 |
| People available | 1 | 1 |
| Collected firewood | 4 | 0 |
| Collected branches | 4 | 0 |
| Fire and coals | 0 | 1 |
| Firesticks | 1 | 1 |

Table 4 Minimum enabling marking and final marking for the places enabling or connecting (highlighted in grey) the Subprocess 4 Collect Small and Large Calcrete Blocks with other subprocesses of the A. coranica model with one available maker

| **Place** | **Initial marking** | **Final marking** | |  |
| --- | --- | --- | --- | --- |
| Start4 | 1 | | 0 | |
| People available | 1 | | 1 | |
| Collected small blocks | 0 | | 1 | |
| Collected large blocks | 0 | | 1 | |
| Large blocks needed | 1 | | 0 | |
| Small blocks needed | 1 | | 0 | |
|  |  | |  | |
|  |  | |  | |
|  |  | |  | |

Table 5 Minimum enabling marking and final marking for the places enabling or connecting (highlighted in grey) the Subprocess 5 Prepare Bulbs with other subprocesses of the A. coranica model with one available maker

| **Place** | **Initial marking** | **Final marking** |
| --- | --- | --- |
| Start5 | 1 | 0 |
| People available | 1 | 1 |
| Bulbs | 4 | 0 |
| Fleshy scales | 0 | 8 |
| Start6 | 0 | 1 |
| Knives | 1 | 1 |
| Bulb remains | 0 | 4 |
| Outer scales | 0 | 4 |

Table 6 Minimum enabling marking and final marking for the places enabling or connecting (highlighted in grey) the Subprocess 6 Heat Selected Scales with other subprocesses of the A. coranica model with one available maker

| **Place** | **Initial marking** | **Final marking** |
| --- | --- | --- |
| Start6 | 1 | 0 |
| People available | 1 | 1 |
| Fire and coals | 1 | 1 |
| Fleshy scales | 8 | 0 |
| Collected large blocks | 1 | 1 |
| Scales on large block | 0 | 8 |
| Coals | 0 | 1 |
| # First scales | 1 | 0 |
| Ready to dust | 0 | 1 |

Table 7 Minimum enabling marking and final marking for the places enabling or connecting (highlighted in grey) the Subprocess 7 Pound with other subprocesses of the A. coranica model with one available maker

| **Place** | **Initial marking** | **Final marking** |
| --- | --- | --- |
| Scales on large block | 8 | 0 |
| People available | 1 | 1 |
| Collected small blocks | 1 | 1 |
| # Scales available for kneading | 0 | 8 |
| Scales ready for kneading | 0 | 8 |
| All scales pounded | 0 | 1 |

Table 8 Minimum enabling marking and final marking for the places enabling or connecting (highlighted in grey) the Subprocess 8 Knead with other subprocesses of the A. coranica model with one available maker

| **Place** | **Initial marking** | **Final marking** | |
| --- | --- | --- | --- |
| # Scales available for kneading | 1 | 0 |  |
| People available | 1 | 1 |  |
| Scales ready for kneading | 1 | 0 |  |
| Fire and coals | 1 | 1 |  |
| All scales pounded | 1 | 0 |  |
| Adhesive | 0 | 1 |  |

Table 9 Minimum enabling marking and final marking for the places enabling or connecting (highlighted in grey) the Subprocess 1 Dig Roots with other subprocesses of the O. schinzii model with one available maker

| **Place** | **Initial marking** | **Final marking** | |
| --- | --- | --- | --- |
| Start1 | 1 | 0 |  |
| People available | 1 | 1 |  |
| Extracted roots | 0 | 4 |  |
| Collecting roots done | 0 | 1 |  |
| *O. schinzii* bush | 1 | 1 |  |
| Digging sticks | 1 | 1 |  |
| Roots needed | 4 | 0 |  |
| Sand | 0 | 3 |  |

Table 10 Minimum enabling marking and final marking for the places enabling or connecting (highlighted in grey) the Subprocess 2 Collect Firewood with other subprocesses of the O. schinzii model with one available maker

| **Place** | **Initial marking** | **Final marking** | |
| --- | --- | --- | --- |
| Start2 | 1 | 0 |  |
| People available | 1 | 1 |  |
| Combretum branches | 0 | 4 |  |
| T. sericea branches | 0 | 4 |  |
| Collecting firewood done | 0 | 1 |  |
| Combretum needed | 4 | 0 |  |
| T. sericea needed | 4 | 0 |  |
|  |  |  |  |
|  |  |  |  |

Table 11 Minimum enabling marking and final marking for the places enabling or connecting (highlighted in grey) the Subprocess 3 Light Fire with other subprocesses of the O. schinzii model with one available maker

| **Place** | **Initial marking** | **Final marking** | |
| --- | --- | --- | --- |
| Start3 | 1 | 0 |  |
| People available | 1 | 1 |  |
| T. sericea branches | 4 | 0 |  |
| Combretum branches | 4 | 0 |  |
| Fire and coals | 0 | 1 |  |
| Start6 | 0 | 1 |  |
| Firesticks | 1 | 1 |  |

Table 12 Minimum enabling marking and final marking for the places enabling or connecting (highlighted in grey) the Subprocess 4 Make Applicator with other subprocesses of the O. schinzii model with one available maker

| **Place** | **Initial marking** | **Final marking** | |
| --- | --- | --- | --- |
| Start4 | 1 | 0 |  |
| People available | 1 | 1 |  |
| Applicator | 0 | 1 |  |
| Start8 | 0 | 1 |  |
| Knives | 1 | 1 |  |
| *G. flava* branch | 1 | 0 |  |

Table 13 Minimum enabling marking and final marking for the places enabling or connecting (highlighted in grey) the Subprocess 5 Root Preparation with other subprocesses of the O. schinzii model with one available maker

| **Place** | **Initial marking** | **Final marking** | |
| --- | --- | --- | --- |
| Start5 | 1 | 0 |  |
| People available | 1 | 1 |  |
| Extracted roots | 4 | 0 |  |
| Roots with slits | 0 | 4 |  |
| Start7 | 0 | 1 |  |
| Knives | 1 | 1 |  |

Table 14 Minimum enabling marking and final marking for the places enabling or connecting (highlighted in grey) the Subprocess 6 Burn and Crush Grass with other subprocesses of the O. schinzii model with one available maker

| **Place** | **Initial marking** | **Final marking** | |
| --- | --- | --- | --- |
| Start6 | 1 | 0 |  |
| People available | 1 | 1 |  |
| Black powder | 0 | 4 |  |
| Fire and coals | 1 | 1 |  |
| Grass needed | 4 | 0 |  |

Table 15 Minimum enabling marking and final marking for the places enabling or connecting (highlighted in grey) the Subprocess 7 Heat Roots with other subprocesses of the O. schinzii model with one available maker

| **Place** | **Initial marking** | **Final marking** | |
| --- | --- | --- | --- |
| Start7 | 1 | 0 |  |
| People available | 1 | 1 |  |
| Fire and coals | 1 | 1 |  |
| Roots with slits | 4 | 0 |  |
| Roots with latex | 0 | 4 |  |
| Coals | 0 | 1 |  |

Table 16 Minimum enabling marking and final marking for the places enabling or connecting (highlighted in grey) the Subprocess 8 Dip and Mix Latex with other subprocesses of the O. schinzii model with one available maker

| **Place** | **Initial marking** | **Final marking** | |
| --- | --- | --- | --- |
| Start8 | 1 | 0 |  |
| People available | 1 | 1 |  |
| Roots with latex | 0 | 4 |  |
| Applicator | 1 | 1 |  |
| Black powder | 4 | 0 |  |
| Glue carrier | 1 | 1 |  |
| Adhesive | 0 | 1 |  |

**Section b). Variables for the A. coranica and O. Schinzii models used to calculate the reachability graphs (Table 17).**

Table 17 Variables for the A. coranica and O. Schinzii models used to calculate the reachability graphs.

| **Model** | **Variables** | **Values** | **Description** |
| --- | --- | --- | --- |
| ***A. coranica*** | *d, c, b, a, h* | 1 | Makers involved in subprocesses |
|  | *nfw, nbr, nb* | 4 | Firewood and branches needed |
|  | *nb* | 4 | Bulbs needed |
|  | *nbs, nbl* | 1 | Small and Large blocks needed |
|  | *x* | 1 | Number of scales put first on coals |
|  | *sb* | 2 | Scales per bulb |
|  | *fs, k, ds* | *p* | Tools from the toolkit |
|  | *p* | 1 to 11 | People available |
| ***O. schinzii*** | *d, c, a, q, m* | 1 | Makers involved in subprocesses |
|  | *nts, nco* | 4 | Firewood and branches needed (T. sericea and Combretum) |
|  | *nr* | 4 | Roots needed |
|  | *ng* | 4 | Grass needed |
|  | *gc* | 1 | Glue carrier |
|  | *fs, k, ds* | *p* | Tools from the toolkit |
|  | *p* | 1 to 11 | People available |
